# Supplementary material for: Inhibiting translation elongation by reducing eIF5A activity induces feedback inhibition of initiation, limiting tumour cell proliferation
Source: Nat Commun. 2025 Dec 13;16:11486. doi: 10.1038/s41467-025-66531-z (PMC12749925; doi:10.1038/s41467-025-66531-z)
Supplement: Supplementary file 1 — Supplementary Information [file 41467_2025_66531_MOESM1_ESM.pdf]

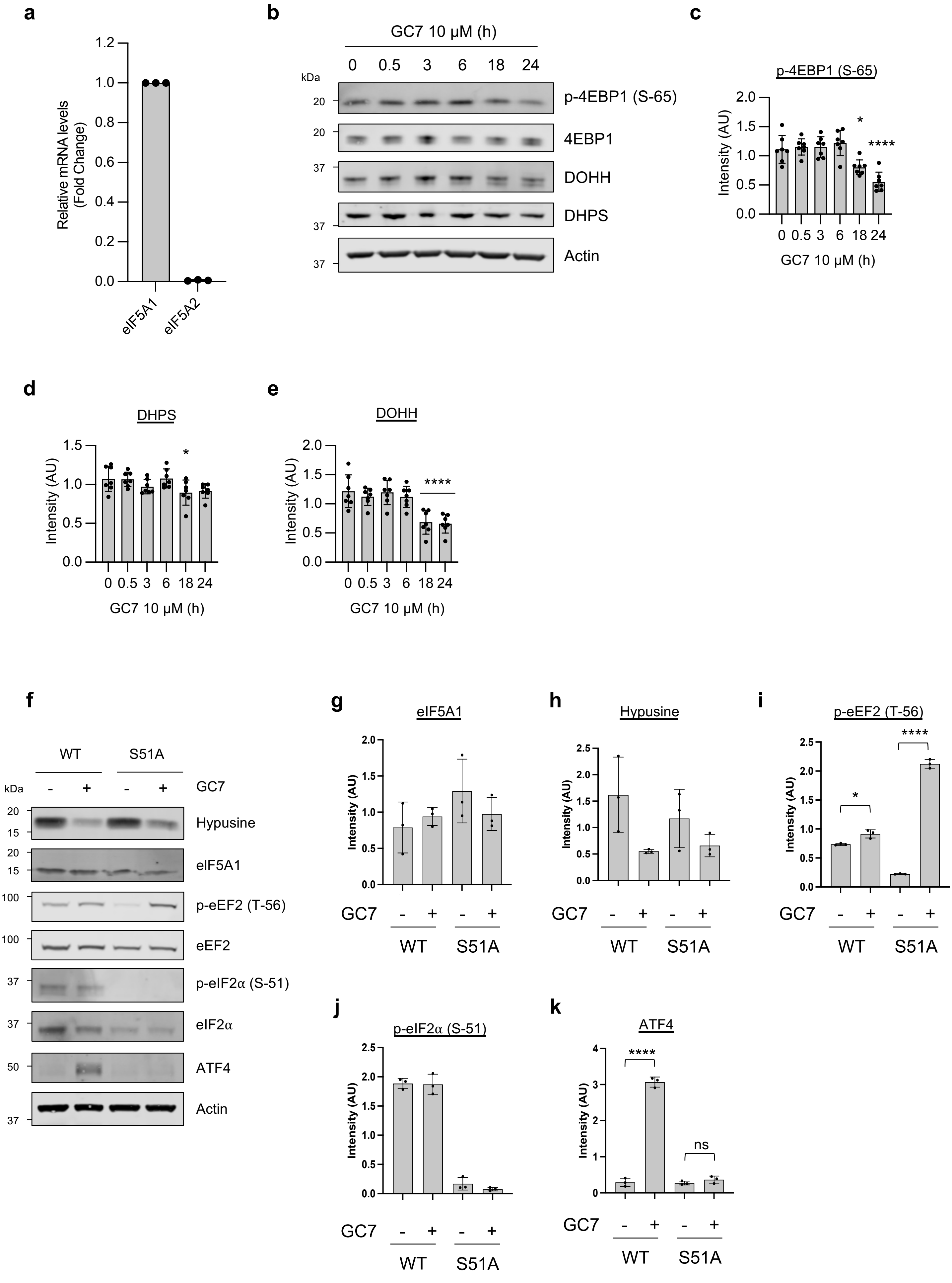

Supplementary Figure 1. Signalling response of A549 and MEFs following treatment with GC7.

Supplementary Figure 1. Signalling response of A549 and MEFs following treatment with GC7.

(a) RT-qPCR was used to determine relative eIF5A1 and eIF5A2 mRNA levels in A549 cells. All error bars represent means ± SD (n=3 independent experiments) and are plotted with individual values **(b)** Representative western blots for the indicated targets in A549 cells treated with 10 µM GC7 for the indicated time points, in parallel to Fig 1a. **(c – e)** Densitometry of (b) p-4EBP1 (c) DHPS and (d) DOHH from (a). Error bars represent means ± SD (n=7 independent experiments) and are plotted with individual values. Statistical analysis was carried out using one-way ANOVA with Dunnett's multiple comparisons test (* = $p<0.05$ and **** = $p<0.0001$) relative to the untreated sample. **(f)** Representative western blots for the indicated targets in WT and S51A mutant MEFs treated with 80 µM GC7 for 24 hours. **(g - k)** Densitometry of the indicated proteins from (E). Error bars represent means ± SD (n = 3 independent experiments) and are plotted with individual values. Statistical analysis was carried out using one-way ANOVA with Tukey's multiple comparisons test (* = $p<0.05$, ** = $p<0.01$, *** = $p<0.001$, **** = $p<0.0001$). Source data are provided within the Source Data file.

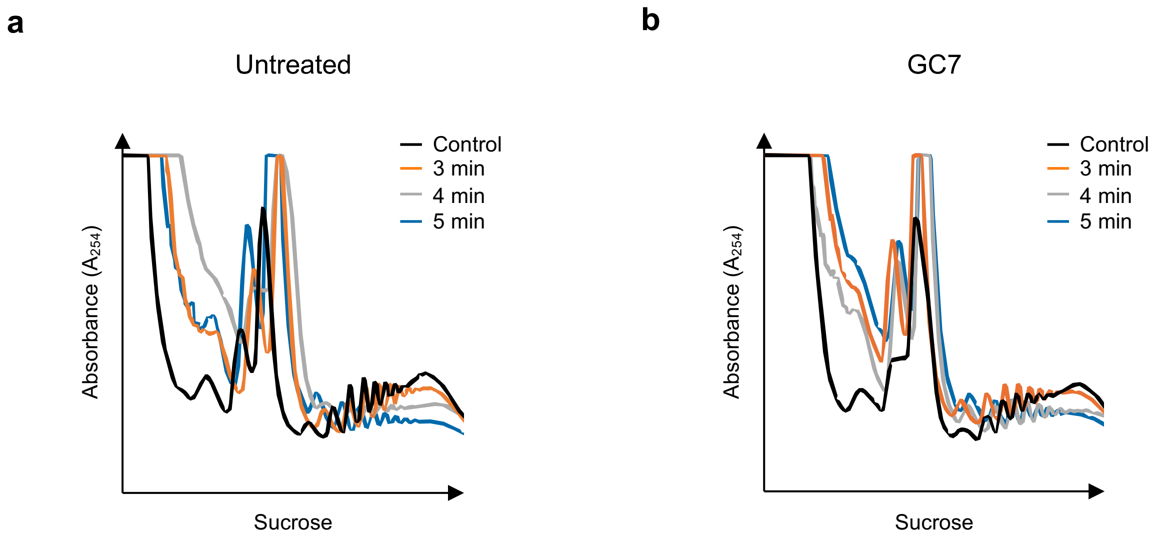

Supplementary Figure 2. Representative polysome profiles of ribosome run-off assay.
(a and b) Representative polysome profiles of A549 cells either (a) untreated or (b) treated with 10 μM GC7 for 3 hours. All cells were treated with harringtonine for either 3, 4 or 5 minutes, and area under polysomes was used to infer ribosome run-off rates (quantified in Fig. 1i). Source data are provided within the Source Data file.

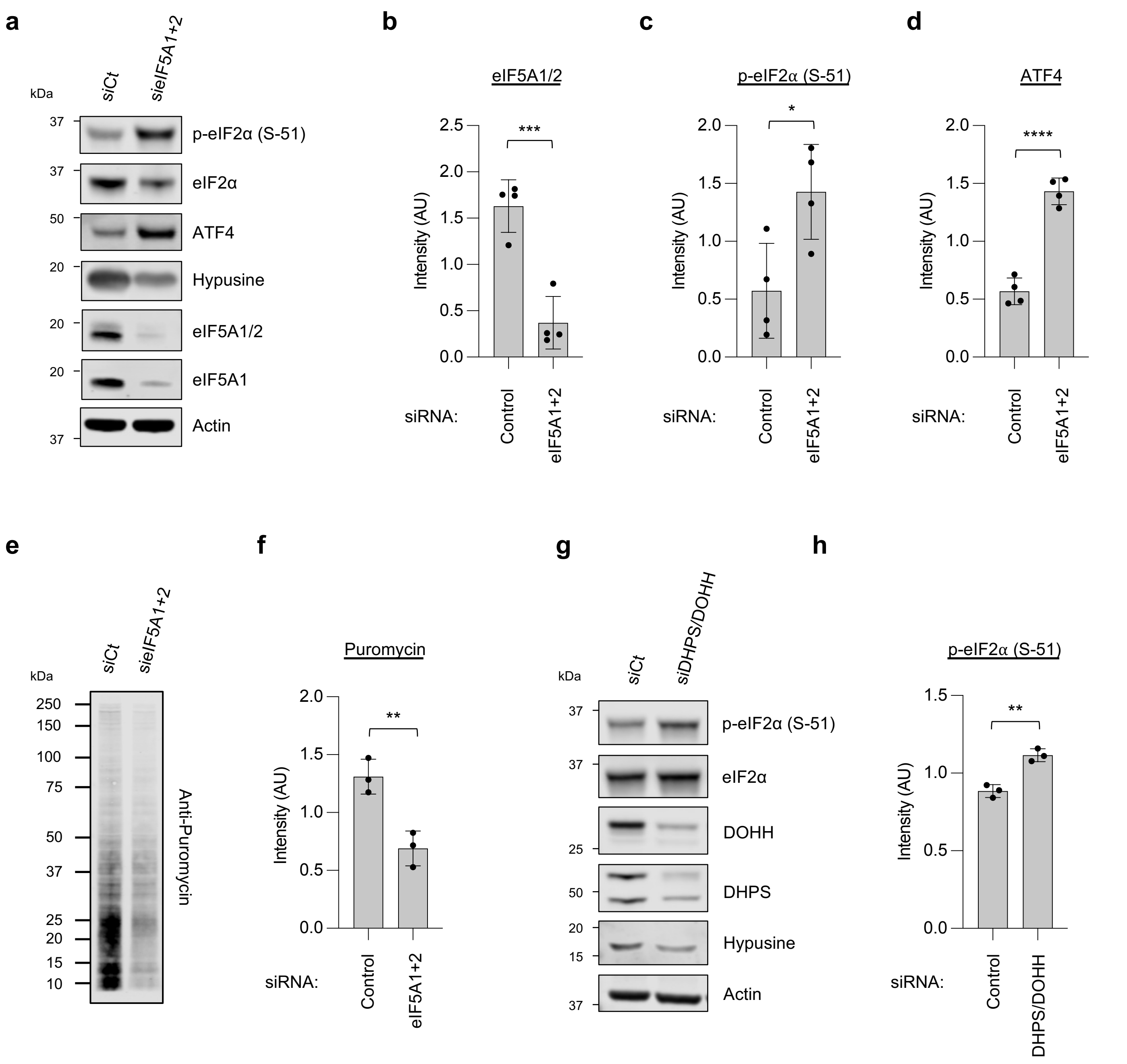

Supplementary Figure 3. Knockdown of eIF5A1 and eIF5A2 recapitulate the effects of GC7 treatment in A549 cells

(a) Representative western blots for the indicated proteins in A549 cells following transfection with either a control non-targeting siRNA or siRNAs specific for both eIF5A1 and 2. **(b - d)** Densitometry for the proteins presented in (a). Error bars represent means ± SD (n=4 independent experiments) and are plotted with individual values. **(e)** Representative western blot analysis of puromycin incorporation in A549 cells following transfection with either a control non-targeting siRNA or siRNAs specific for both eIF5A1 and 2. **(f)** Densitometry for puromycin incorporation presented in (e). Error bars represent means ± SD (n=3 independent experiments) and are plotted with individual values. **(g)** Representative western blots for the indicated proteins in A549 cells following transfection with either a control non-targeting siRNA or siRNAs specific for both DHPS and DOHH. **(h)** Densitometry analysis of p-eIF2α from (g). Error bars represent means ± SD (n=3 independent experiments) and are plotted with individual values. All statistical analysis was carried out using two-tailed unpaired student's t test (* = $p<0.05$, ** = $p<0.01$, *** = $p<0.001$, **** = $p<0.0001$). Source data are provided within the Source Data file.

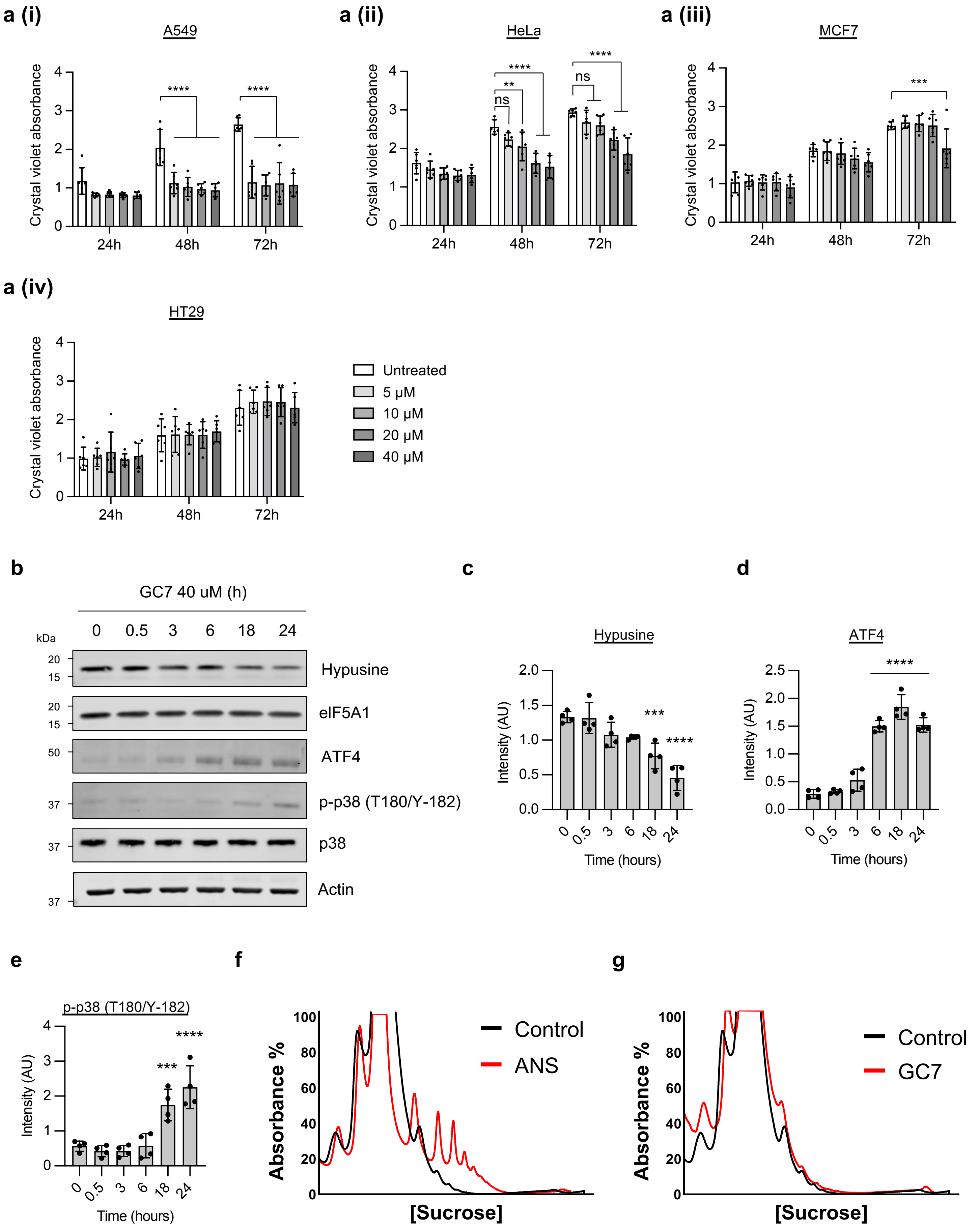

Supplementary Figure 4. GC7 induces minimal ribosome collisions in HeLa cells.

Supplementary Figure 4. GC7 induces minimal ribosome collisions in HeLa cells.

(a) Crystal violet staining was carried out to infer cell number in **(i)** A549, **(ii)** HeLa, **(iii)** MCF7 and **(iv)** HT29 cells treated with 5 µM, 10 µM, 20 µM or 40 µM GC7 and at 24, 48 and 72 hours. Error bars represent means ± SD (n=6 independent experiments) and are plotted with individual values. Statistical analysis was carried out using two-way ANOVA with Dunnett's multiple comparisons test (ns = not significant, * = p<0.05, ** = p<0.01, *** = p<0.001 and **** = p<0.0001). Timepoints without indicated p-values were not significant. **(b)** Representative western blots for the indicated proteins in HeLa cells treated with 40 µM GC7 for the indicated time points. **(c - e)** Densitometry of (c) hypusine, (d) ATF4 and (e) p-p38 (T180/Y-182) from time points presented in (b). Error bars represent means ± SD (n=4 independent experiments) and are plotted with individual values. Statistical analysis was carried out using one-way ANOVA with Dunnett's multiple comparisons test (* = p<0.05, *** = p<0.001, **** = p<0.0001) relative to the untreated sample. **(f and g)** Ribosome stalling assay of HeLa cells grown in normal conditions vs cells treated with (f) anisomycin (ANS) or (g) 40 µM GC7 for 3 hours. A single control trace is shown in both f and g as they were part of the same experiment and only separated for the purposes of visualization. Source data are provided within the Source Data file.

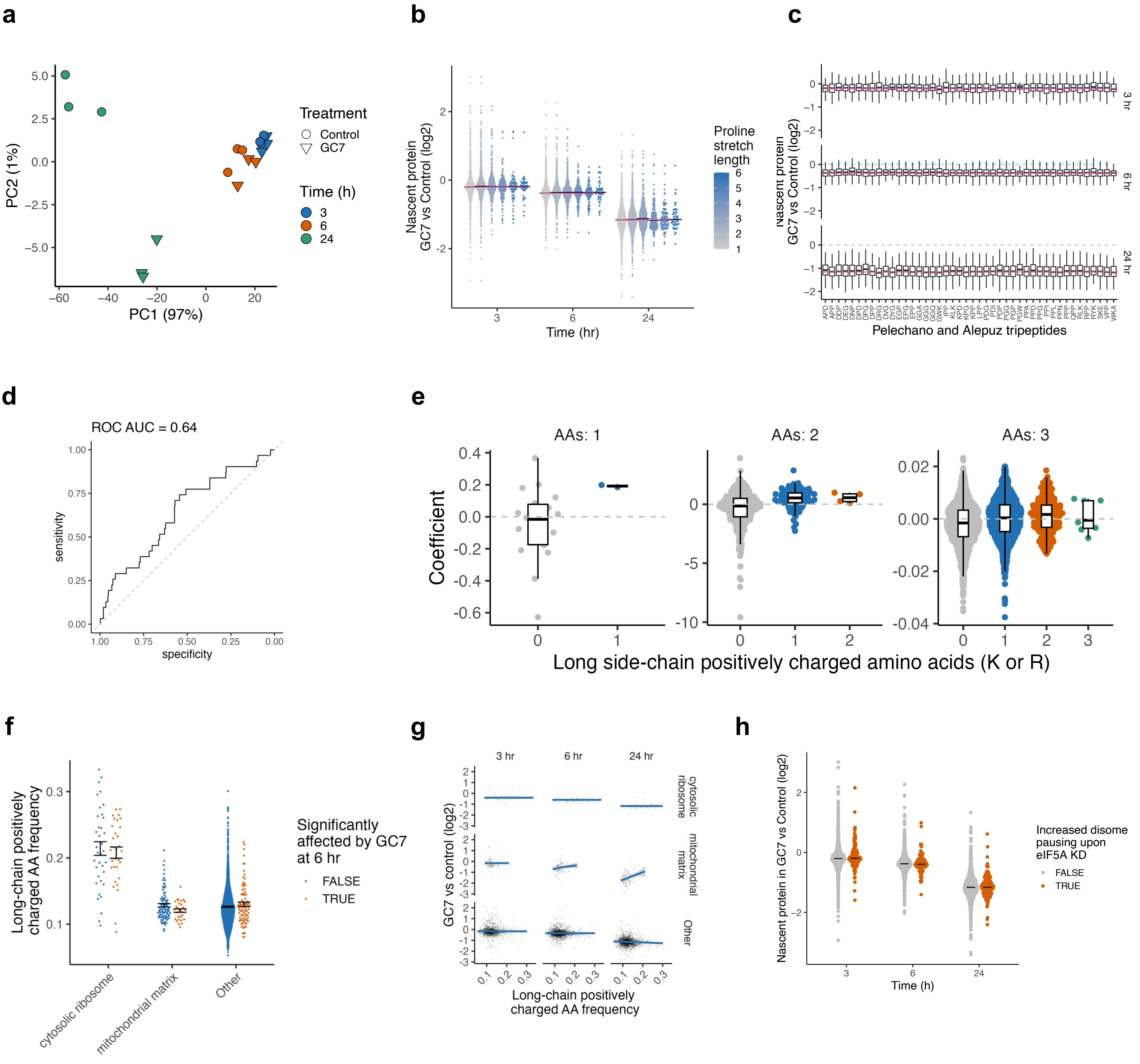

Supplementary Figure 5. Hypusination inhibition does not inhibit translation of specific tri-peptides.

(a) PCA plots showing grouping of samples and replicates (3 independent biological replicates) used for dynamic SILAC analysis (Fig. 2). Percentage values indicate the proportions of variance by the PCs. **(b)** Effect of GC7 on proline translation. Proteins were separated by their longest poly-proline tract. Pink line represents the mean GC7 vs control difference. **(c)** GC7 vs control newly synthesized proteins with tri-peptides previously identified as eIF5A-dependent in Pelechano and Alepuz 2017 [1]. Box extends to the 25th and 75th percentiles. Whiskers extend to range, excluding outliers. Outliers are defined as greater than 1.5 x the interquartile range from the box. Pink line represents the mean GC7 vs control difference. **(d)** Receiver Operator Curve for Ridge Regression model of GC7 effect using amino acid, di-peptide and tri-peptides as features. AUC=area under curve. **(e)** Ridge Regression coefficients for sequences of varying amino acid (AA) length. Data shown is separated by the length of sequence (AAs 1 - 3) and proportion of long side-chain positively charged amino acids (lysines and arginines) within the sequence. AA=Amino acids. Box extends to the 25th and 75th percentiles. Whiskers extend to range, excluding outliers. Outliers are defined as greater than 1.5 x the interquartile range from the box. **(f)** Frequency of lysine and arginine in proteins whose synthesis is affected by GC7 at 6 hours, separated by their functional annotation. **(g)** Frequency of lysine and arginine vs GC7 effect, separated by functional annotation. **(h)** GC7 vs control for proteins with increased disome pausing upon eIF5A knockdown (KD) from Han et al 2020 [2].

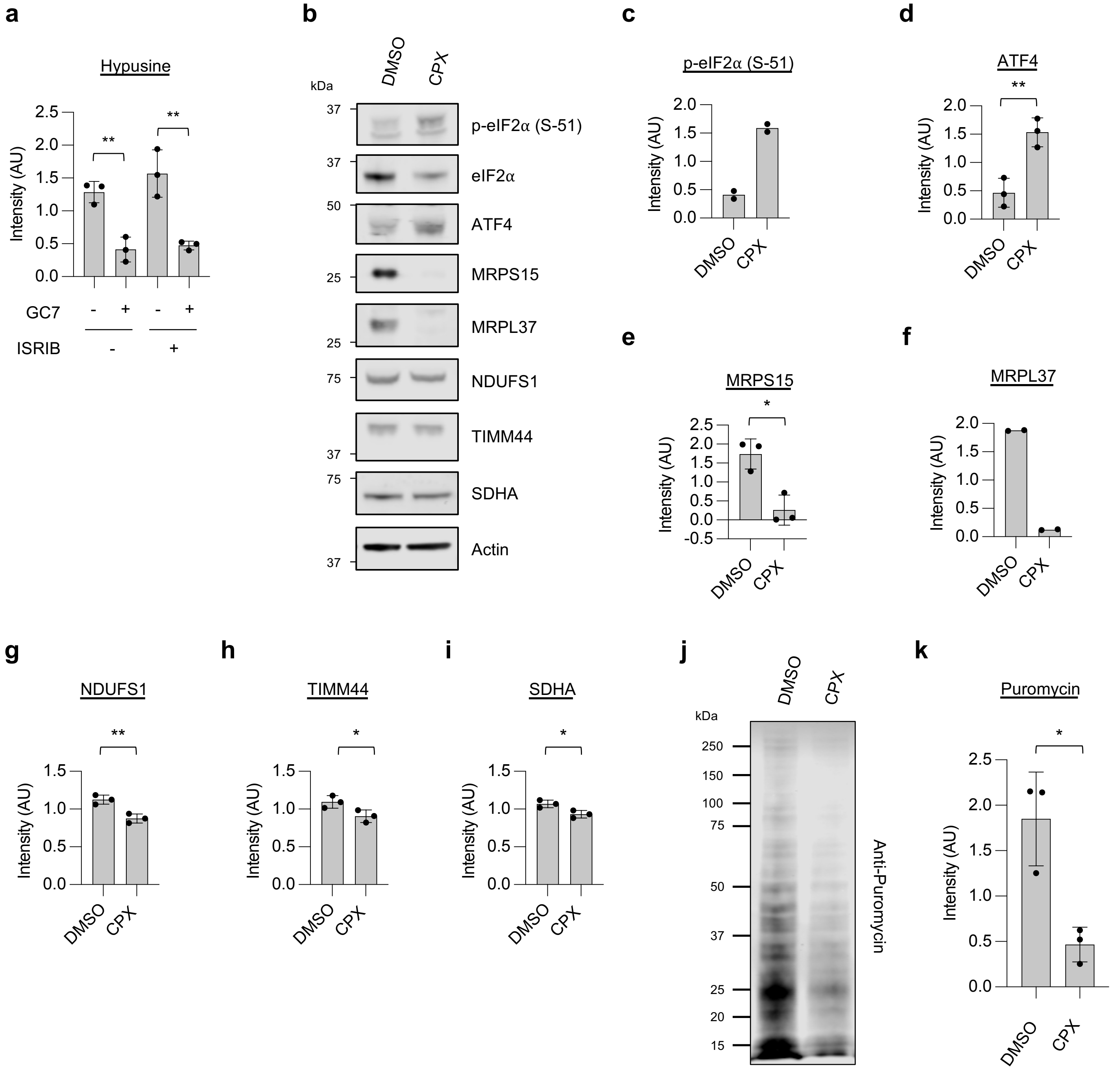

Supplementary Figure 6. Treatment with ISRIB does not impact GC7-depenendent inhibition of hypusination.

(a) Quantification of hypusine presented in Fig. 3a. Error bars represent means ± SD (n=3 independent experiments) and are plotted with individual values. Statistical analysis was carried out using One-Way ANOVA with Tukey's multiple comparisons test (** = p<0.01, *** = p<0.001). **(b)** Representative western blots for the indicated targets in A549 cells treated with 10 μM CPX for 24 hours. **(c – i)** Densitometry of the indicated proteins presented in (b). Error bars represent means ± SD (n=3 independent experiments), expect MRPL37 and p-eIF2α (n=2 independent experiments), and are plotted with individual values. Statistical analysis was carried out using two-tailed unpaired student's t test (* = p<0.05, ** = p<0.01). **(j)** Representative western blot analysis of puromycin incorporation in A549 cells treated with 10 μM CPX for 24 hours. **(k)** Densitometry for puromycin incorporation presented in (j). Error bars represent means ± SD (n=3 independent experiments) and are plotted with individual values. Statistical analysis was carried out using two-tailed unpaired student's t test (* = p<0.05, ** = p<0.01). Source data are provided within the Source Data file.

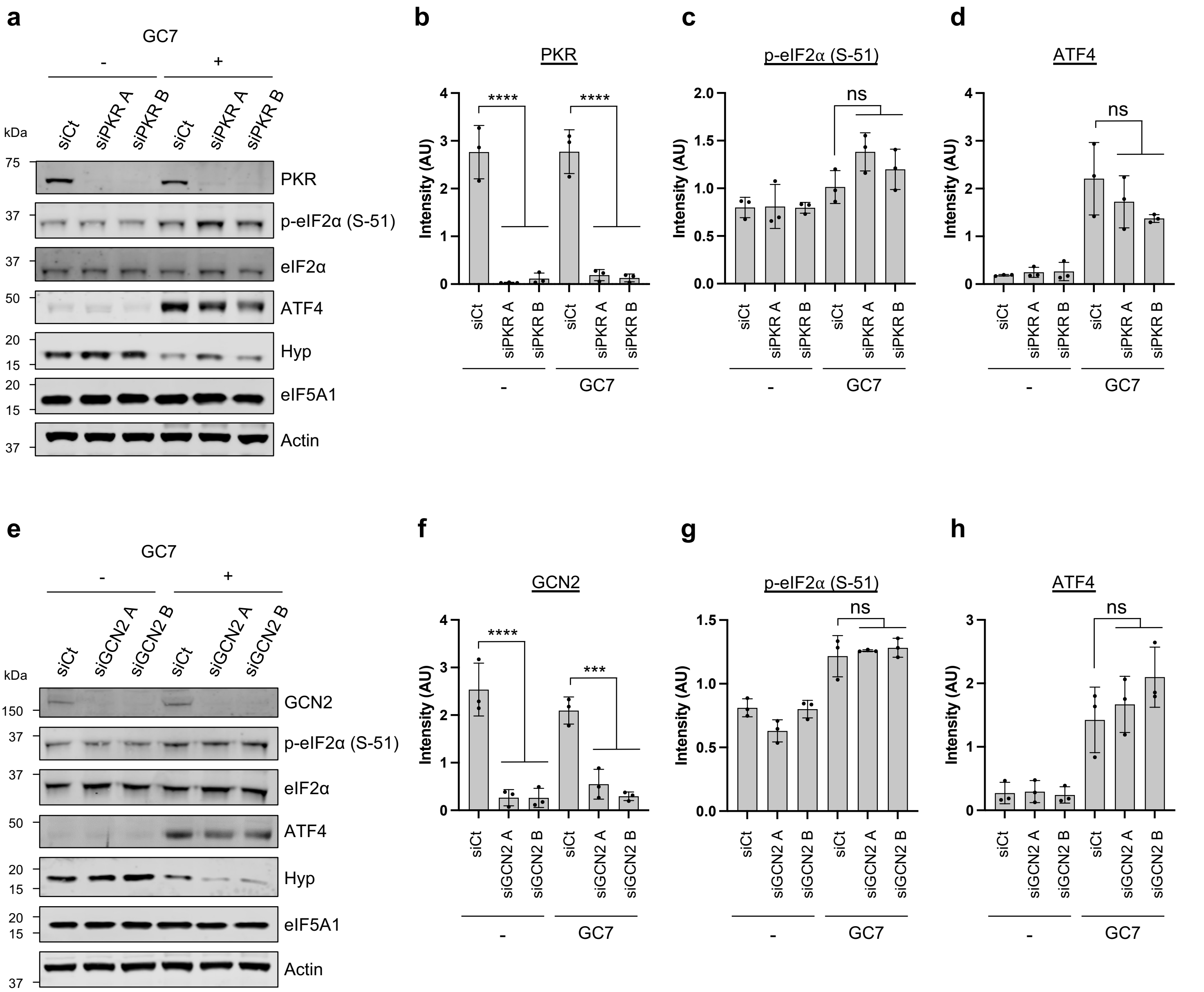

Supplementary Figure 7 – Translation initiation inhibition following eIF5A inhibition is driven by PERK and HRI activation and eIF2α phosphorylation.

(a) Representative western blots for the indicated proteins from A549 cells transfected with siRNAs against PKR and treated with 10 μM of GC7 for 24 hours. **(b - d)** Densitometry of western blots for the indicated proteins from (a). **(e)** Representative western blots for the indicated targets for A549 cells transfected with siRNAs against GCN2 and treated with 10 μM of GC7 for 24 hours. **(f - h)** Densitometry of western blots for the indicated proteins from (e). All error bars represent means ± SD (n=3 independent experiments) and are plotted with individual values Statistical analysis was carried out using One-Way ANOVA with Tukey's multiple comparisons test (ns = not significant, *** = $p<0.001$, **** = $p<0.0001$). Source data are provided within the Source Data file.

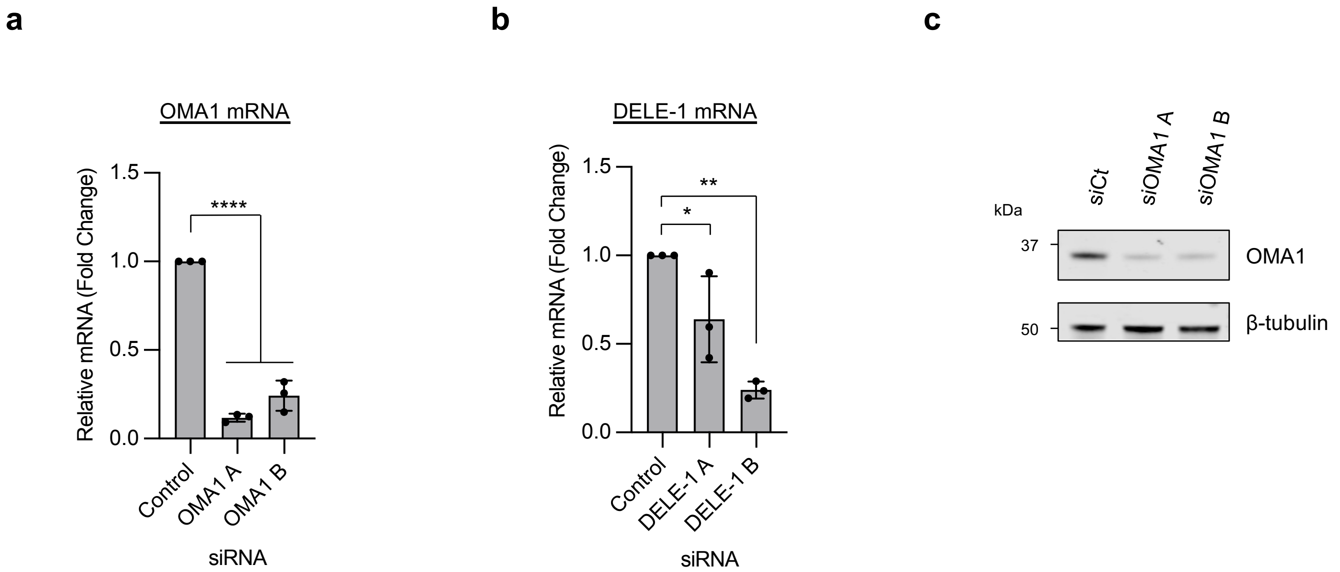

Supplementary Figure 8. Knockdown of DELE-1 and OMA1 using siRNAs in A549 cells.

A549 cells were transfected with a control non-targeting siRNA or two different siRNAs specific for either **(a)** OMA1 or **(b)** DELE-1. RT-qPCR was used to determine the relative mRNA levels. All error bars represent means ± SD (n=3 independent experiments) and are plotted with individual values Statistical analysis was carried out using one-way ANOVA with Dunnett's multiple comparisons test (* = $p<0.05$, ** = $p<0.01$ and **** = $p<0.0001$). **(c)** In parallel to (a), representative western blot for OMA1 protein levels in A549 cells after transfection with the indicated siRNAs. Source data are provided within the Source Data file.

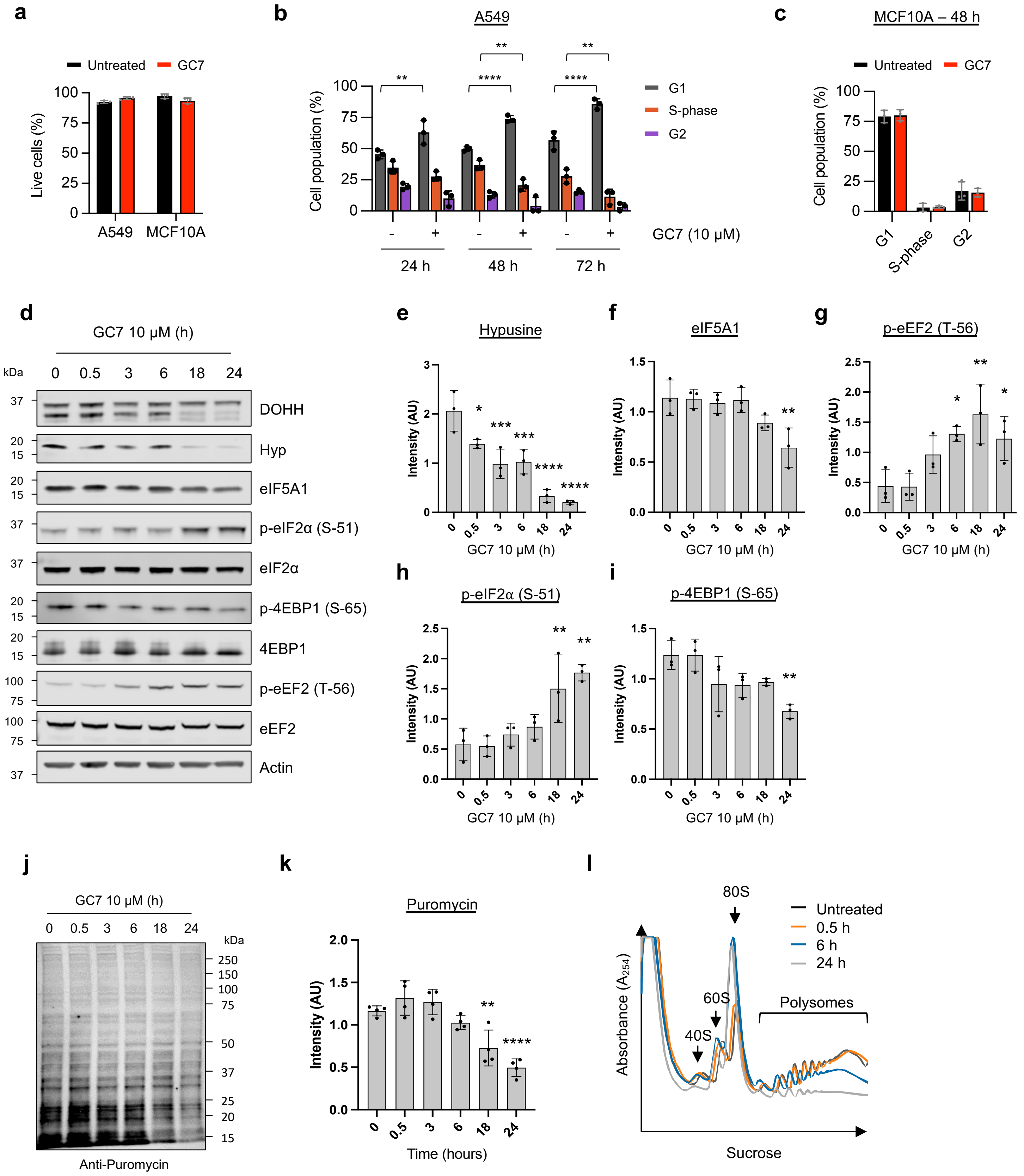

Supplementary Figure 9. Hypusination inhibition leads to sequential elongation and initiation blocks in MCF10A Cells.

Supplementary Figure 9. Hypusination inhibition leads to sequential elongation and initiation blocks in MCF10A Cells.

(a) Quantification of cell viability using annexin V and Draq7 staining of A549 and MCF10A cells treated with 10 μM GC7 for 48 hours. Error bars represent means ± SD (n=3 independent experiments) and are plotted with individual values. **(b)** Cell cycle distribution of A549 cells treated with 10 μM GC7 for the indicated time. DNA was stained using FxCycle violet dye and quantified using the Dean-Jett-Fox model. Error bars represent means ± SD (n=3 independent experiments). Statistical analysis was carried out using two-way ANOVA with Tukey's multiple comparisons test (** = p<0.01, **** = p<0.0001). **(c)** Cell cycle distribution of MCF10A cells treated with 10 μM GC7 for 48 hours. DNA was stained using PI and quantified using the Dean-Jett-Fox model. Error bars represent means ± SD (n=3 independent experiments) and statistical analysis with two-way ANOVA with Sidak's multiple comparisons test (** = p<0.01, *** = p<0.001). **(d)** Representative western blots for the indicated targets in MCF10A cells treated with 10 μM GC7 for the indicated time points. **(e - i)** Quantification of the indicated blots from (a). Error bars represent means ± SD (n=3 independent experiments) and are plotted with individual values. Statistical analysis was carried out using One-Way ANOVA with Dunnett's multiple comparisons test (* = p<0.01, ** = p<0.01, ** = p<0.001, **** = p<0.0001) relative to the untreated sample. **(j)** Representative western blot analysis of puromycin incorporation in MCF10A cells treated with 10 μM GC7 for the indicated time points. **(k)** Quantification of puromycin signal shown in (g). Error bars represent means ± SD (n=4 independent experiments) and are plotted with individual values Statistical analysis was carried out using One-Way ANOVA with Dunnett's multiple comparisons test (** = p<0.01, **** = p<0.0001) relative to the untreated sample. **(l)** Polysome profiles of MCF10A cells untreated or treated with 10 μM GC7 for 0.5, 6 or 24 hours. Source data are provided within the Source Data file.

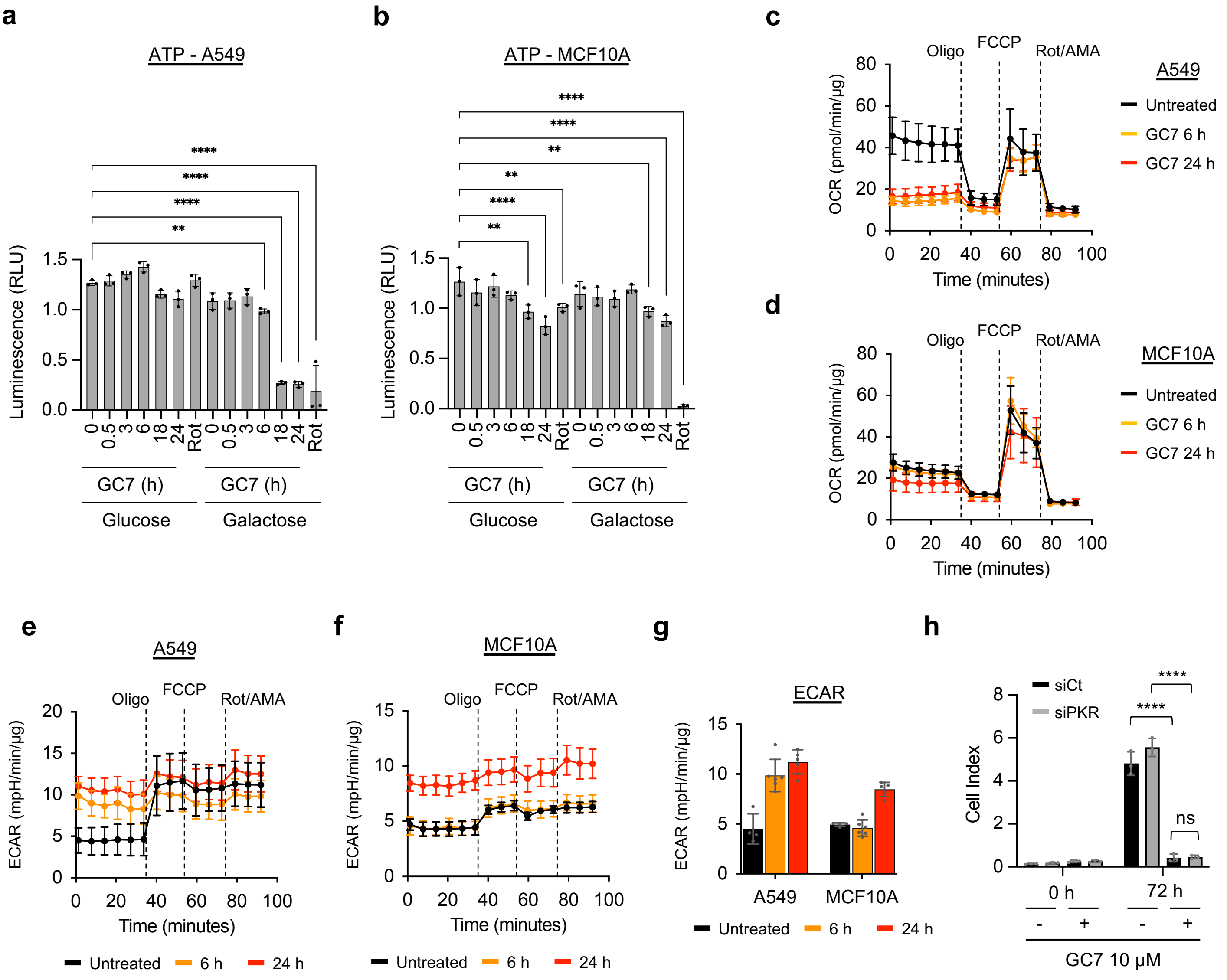

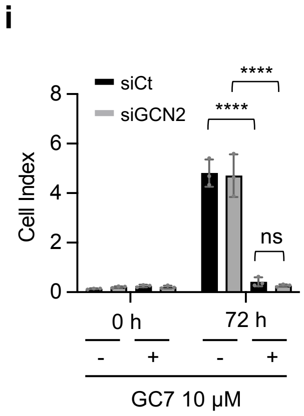

Supplementary Figure 10. A549 cells have dysfunctional mitochondria relative to MCF10A and GC7 inhibits mitochondrial function.
(a and b) ATP assay in A549 (a) and MCF10A (b) cells grown in Glucose or Galactose and treated with 10 μM GC7 for the indicated time points. Rotenone was used as a positive control. Error bars represent means ± SD (n=3 independent experiments). Statistical analysis using One-Way ANOVA with Dunnett's multiple comparisons test (* = p<0.05, ** = p<0.01, **** = p<0.0001). **(c and d)** Oxygen consumption rates of (c) A549 and (d) MCF10A cells pretreated with 10 μM GC7 for 6 or 24 hours. Cells were treated with oligomycin (2 μM), FCCP (500 nM), rotenone and antimycin A (2 μM) at the indicted timepoints. Representative experiment used to generate values in Fig. 6d and 6e. OCR was normalised to total protein and error bars represent means ± SD from technical replicates (A549 untreated n=4, 6 hr and 24 hr n= 6; MCF10A untreated n=5, 6 hr n=6 and 24 hr n= 5). **(e and f)** In parallel to (c and d), extracellular acidification rate (ECAR) of (e) A549 and (f) MCF10A cells pretreated with 10 μM GC7 for 6 or 24 hours. ECAR was normalized to total protein and error bars represent means ± SD from technical replicates (A549 untreated n=4, 6 hr and 24 hr n= 6; MCF10A untreated n=5, 6 hr n=6 and 24 hr n= 5). **(g)** ECAR taken from (e and f). **(h and i)** Cell index from the xCELLigence RTCA instrument of A549 cells transfected with siRNAs specific to (h) PKR or (i) GCN2 and treated with 10 μM GC7 for 72 hours. Error bars represent means ± SD (n=3 independent experiments). Statistical analysis using two-way ANOVA with Tukey's multiple comparisons test (ns = not significant and **** = p<0.0001). Source data are provided within the Source Data file.

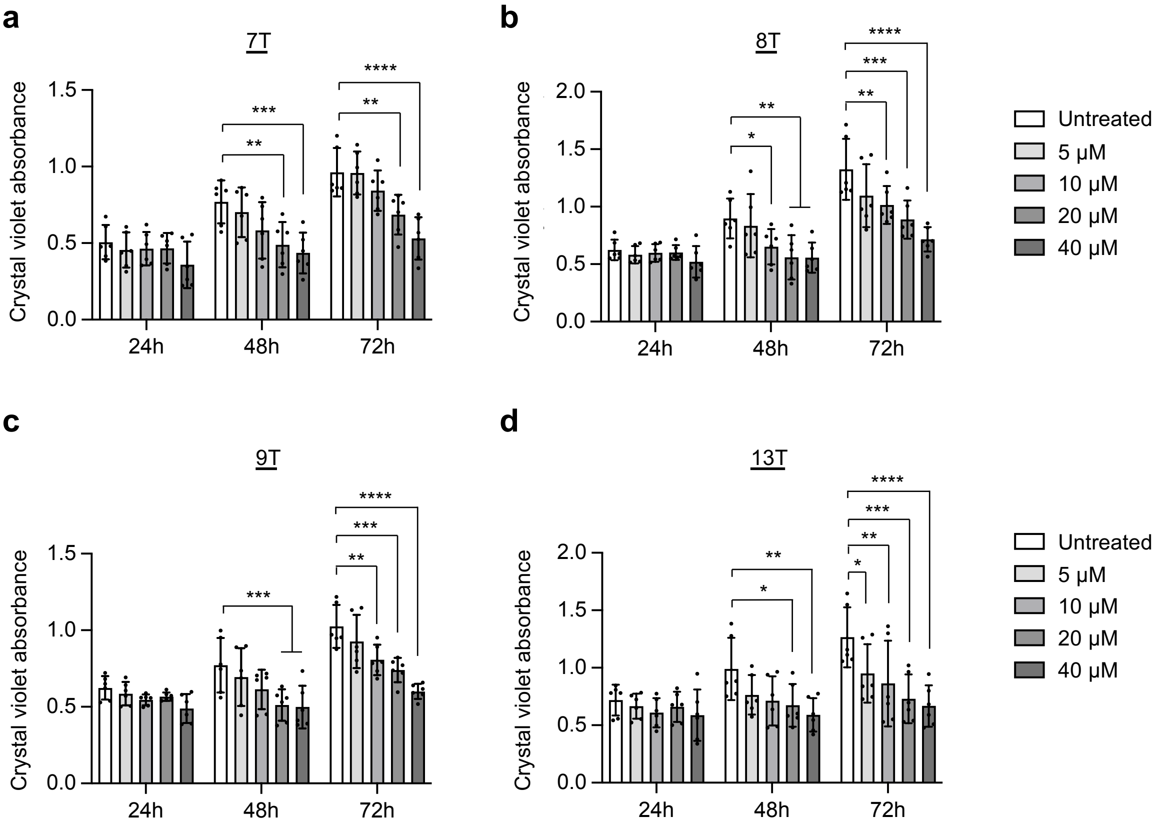

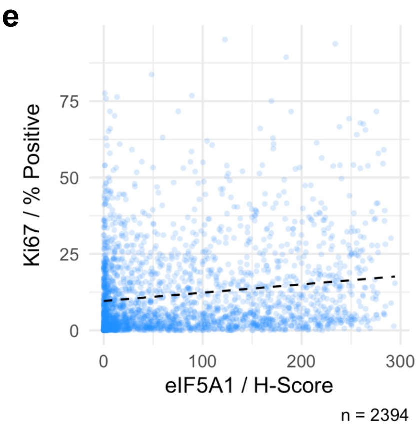

f

	R	P	n
Ki67/%Positive Cells	0.180	2.12×10^{-19}	2,394

Supplementary Figure 11 GC7 dose response and time course in primary mesothelial cell lines

Crystal violet staining was carried out to infer cell number in **(a)** 7T, **(b)** 8T, **(c)** 9T and **(d)** 13T primary mesothelioma cell lines treated with 5 μM, 10 μM, 20 μM or 40 μM GC7 and at 24, 48 and 72 hours. Error bars represent means ± SD (n=6 independent experiments) and are plotted with individual values. Statistical analysis was carried out using two-way ANOVA with Dunnett's multiple comparisons test (* = $p<0.05$, ** = $p<0.01$, *** = $p<0.001$ and **** = $p<0.0001$). Timepoints without indicated p-values were not significant. **(e)** Scatter plot of core level tumour H-Score for IHC staining of eIF5A1 performed on a human primary resected lung adenocarcinoma tissue microarray against Ki67 index. Each point represents one TMA-Core (n = 2,394). **(f)** Correlation coefficient and p-value shown for spearman correlation of eIF5A1 versus Ki67 index. Source data are provided within the Source Data file.

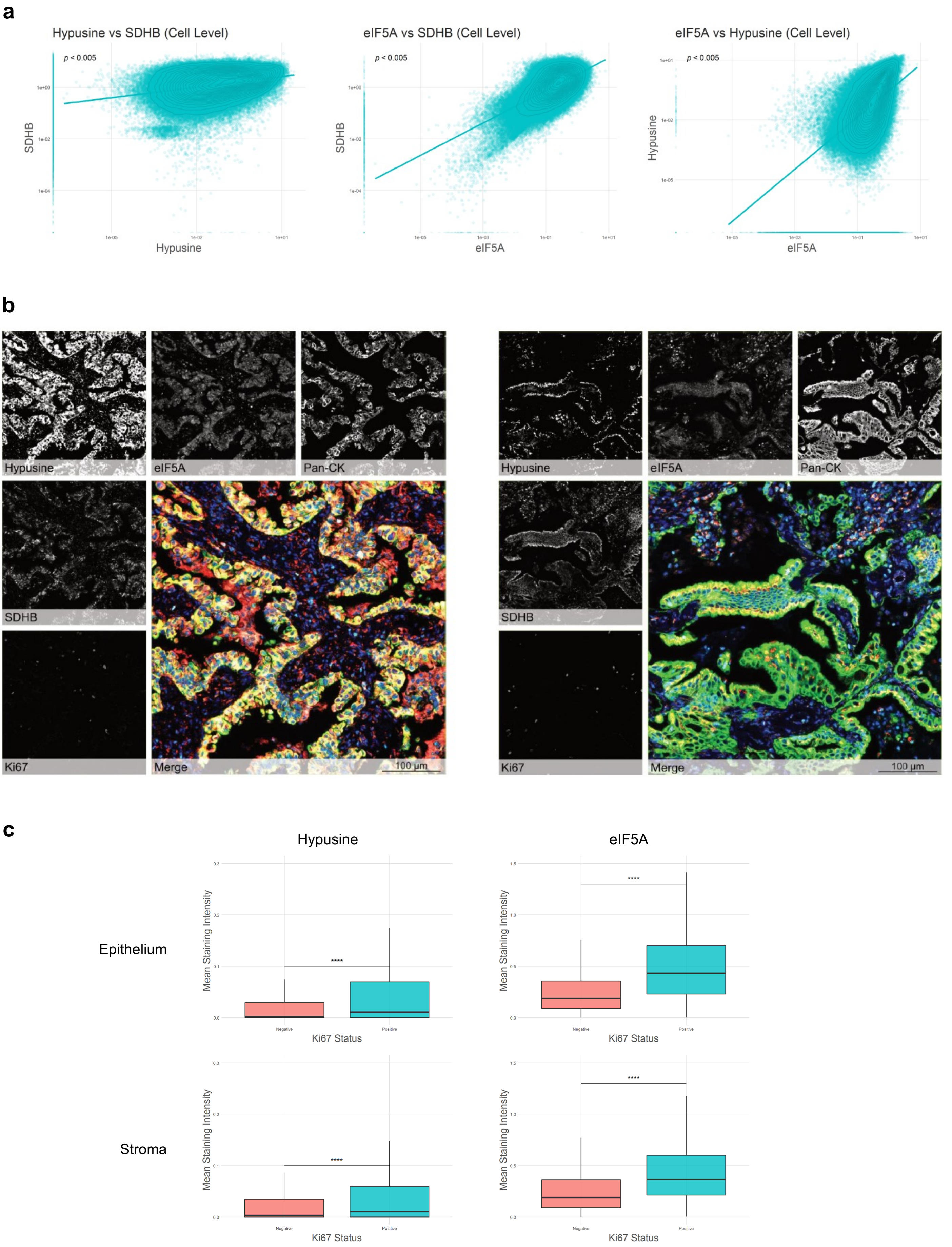

Supplementary Figure 12 – A human primary resected lung adenocarcinoma tissue microarray was stained for eIF5A, Hypusine, SDHB, pan-cytokeratin and Ki67 using multiplexed immunofluorescence.

Supplementary Figure 12 – A human primary resected lung adenocarcinoma tissue microarray was stained for eIF5A, Hypusine, SDHB, pan-cytokeratin and Ki67 using multiplexed immunofluorescence.

(a) Scatter plots of tumour cell level mean intensities of the indicated markers. P-value is derived from Spearmans Rank test. (b) Representative Images from the multiplexed immunofluorescence panel described above. Pan-Cytokeratin (Opal-620), eIF5A (Opal-690), Hypusine (Opal-520), SDHB (Opal-650), Ki67 (Opal-570) and counterstained with DAPI. (C) Box and whisker plots of cell level mean intensities of eIF5A and Hypusine stratified by Ki67 status. Box plots represent median values with hinges corresponding to the first and third quartiles and whiskers extending to no further than 1.5* the interquartile range from the hinge. P-value from pairwise t-test.

Supplementary Table 1. Compounds used in this study

Compound	Supplier	Catalogue Number
GC7	Merck	CAS 150333-69-0
ISRIB	Sigma-Aldrich	SML0843
Ciclopirox	Sigma-Aldrich	SML2011
Harringtonine	Santa Cruz	sc-204771
Anisomycin	Sigma-Aldrich	#A9789
Rotenone	Merck	R8875
Puromycin	ThermoFisher	A1113803
Hydrocortisone	Sigma-Aldrich	Cat. #H0888
Insulin	Sigma-Aldrich	Cat. #I9278
EGF	Peprotech	Cat. #AF-100-15
Cholera toxin	Sigma-Aldrich	Cat. #C8052
Hygromycin	Thermo Fisher	Cat. #10687010
Cycloheximide	Sigma-Aldrich	Cat. #C7698
Complete EDTA-free protease inhibitor cocktail	Roche	Cat. #11836170001
PhosSTOP phosphatase inhibitors	Roche	Cat. # 04906837001
Annexin V-FITC	Thermo Fisher	Cat. #A13199
DRAQ7	Thermo Fisher	Cat. #15106
Annexin binding buffer	BD Biosciences	Cat. #556454
L-[35S]-methionine (1000 Ci/mmol)	Hartmann Analytic	Cat. #SCIS-103

Supplementary Table 2. Antibodies used for western blotting in this study

Antibody	Supplier	Catalogue Number	Dilution used
Hypusine (Hpu98)	Creative Biolabs	PABL-582	1:1000
eIF5A1	Abcam	ab32443	1:1000
EIF5A1/EIF5A2	Proteintech	17069-1-AP	1:1000
DOHH	Abcam	ab197587	1:1000
DHPS	Abcam	ab190266	1:1000
p-eIF2α (Ser51)	Abcam	Ab32157	1:1000
eIF2α	Cell Signaling	#9722S	1:1000
ATF4	Cell Signaling	#11815	1:1000
ATF4	Abcam	ab270980	1:1000
Phospho-4E-BP1 (Ser65)	Cell Signaling	#9451S	1:1000
4E-BP1	Cell Signaling	#9644S	1:1000
Phospho-eEF2 (Thr56)	Cell Signaling	#2331S	1:1000
eEF2	Santa Cruz	sc-166415	1:1000
Actin	Sigma-Aldrich	A5441	1:1000
β-tubulin	Cell Signaling	#2146	1:1000
Ndufs1	Abcam	ab169540	1:1000
TIM44	Abcam	ab194829	1:1000
TOM20	Cell Signaling	424065	1:1000
SDHA	Abcam	ab14715	1:1000
SDHB	Abcam	ab14714	1:1000
MRPL37	Proteintech	15190-1-AP	1:1000
MRPS15	Abcam	ab242120	1:1000
RPS6	Cell Signaling	#2217S	1:1000
RPS25	Abcam	ab254671	1:1000
HRI	Invitrogen	#702551	1:1000
PERK (C33E10)	Cell Signaling	#3192S	1:1000
PKR	Cell Signaling	#12297S	1:1000
GCN2	Cell Signaling	#3302S	1:1000
Puromycin (clone 12D10)	Merck	MABE343	1:10,000
Phospho-p38 MAPK (Thr180/Tyr182)	Cell Signaling	#4511S	1:1000
p38 MAPK	Cell Signaling	#9212S	1:1000
Secondary anti-mouse IgG (H+L) (DyLight 800)	Cell Signaling	#5257	1:15,000
Secondary anti-mouse IgG (H+L) (DyLight 680)	Cell Signaling	#5470	1:15,000
Secondary anti-rabbit IgG (H+L) (DyLight 680)	Cell Signaling	#5366	1:15,000
Secondary anti-rabbit IgG (H+L) (DyLight 800)	Cell Signaling	#5151	1:15,000

Supplementary Table 3: Primers used in this study

**Primers**	**Supplier**	**Sequence (5'-3')**
OMA1 FWR	SIGMA ALDRICH	AACCCAAGATGCCAGAATGGT
OMA1 REV	SIGMA ALDRICH	AATGCTTCGTGCTGAGTTTGA
DELE1 FWR	SIGMA ALDRICH	CTAGCCTCTGGAGGGTGACT
DELE1 REV	SIGMA ALDRICH	ATAGGGTGTTCGGGGAGACA

Supplementary Table 4. siRNAs used in this study

siRNAs	Supplier	Catalogue Number
EIF5A1	Horizon Discovery	J-015739-05-0002
EIF5A2	Horizon Discovery	J-019191-09-0002
HRI A	Horizon Discovery	J-005007-05-0002
HRI B	Horizon Discovery	J-005007-06-0002
PKR A	Horizon Discovery	J-003527-10-0002
PKR B	Horizon Discovery	J-003527-11-0002
PERK A	Horizon Discovery	J-004883-10-0002
PERK B	Horizon Discovery	J-004883-11-0002
GCN2 A	Horizon Discovery	J-005314-07-0002
GCN2 B	Horizon Discovery	J-005314-08-0002
OMA1 A	Horizon Discovery	J-008662-05-0002
OMA1 B	Horizon Discovery	J-008662-06-0002
DELE1 A	Horizon Discovery	J-021022-17-0002
DELE1 B	Horizon Discovery	J-021022-18-0002
DHPS	Horizon Discovery	J-006670-05-0002
DOHH	Horizon Discovery	J-014712-09-0002

Supplementary References

1. Pelechano, V. & Alepuz, P. eIF5A facilitates translation termination globally and promotes the elongation of many non polyproline-specific tripeptide sequences. *Nucleic Acids Res* **45**, 7326-7338 (2017).

2. Han, P. *et al.* Genome-wide Survey of Ribosome Collision. *Cell Rep* **31**, 107610 (2020).
