# Supplementary material for: Inhibiting translation elongation by reducing eIF5A activity induces feedback inhibition of initiation, limiting tumour cell proliferation
Source: Nat Commun. 2025 Dec 13;16:11486. doi: 10.1038/s41467-025-66531-z (PMC12749925; doi:10.1038/s41467-025-66531-z)
Supplement: Supplementary file 2 — Description of Additional Supplementary Files [file 41467_2025_66531_MOESM2_ESM.docx]

Supplementary Data 1. Quantification of protein expression using dynamic SILAC.

Supplementary Data 2. Tumour H score for IHC of eIF5A1.

Supplementary Data 3. TMA Intensities for Hypusine, eIF5A, SDHB and Ki67.
