# Supplementary material for: Inhibiting translation elongation by reducing eIF5A activity induces feedback inhibition of initiation, limiting tumour cell proliferation
Source: Nat Commun. 2025 Dec 13;16:11486. doi: 10.1038/s41467-025-66531-z (PMC12749925; doi:10.1038/s41467-025-66531-z)
Supplement: Supplementary file 6 — Reporting Summary [file 41467_2025_66531_MOESM6_ESM.pdf]

nature portfolio

Corresponding author(s): Anne E. Willis, Robert F. Harvey and Aristeidis P. Sfakianos.

Last updated by author(s): 14-10-2025

Reporting Summary

Nature Portfolio wishes to improve the reproducibility of the work that we publish. This form provides structure for consistency and transparency in reporting. For further information on Nature Portfolio policies, see our Editorial Policies and the Editorial Policy Checklist.

Statistics

For all statistical analyses, confirm that the following items are present in the figure legend, table legend, main text, or Methods section.

n/a	Confirmed	
☐	☒	The exact sample size (*n*) for each experimental group/condition, given as a discrete number and unit of measurement
☐	☒	A statement on whether measurements were taken from distinct samples or whether the same sample was measured repeatedly
☐	☒	The statistical test(s) used AND whether they are one- or two-sided<br>*Only common tests should be described solely by name; describe more complex techniques in the Methods section.*
☐	☒	A description of all covariates tested
☐	☒	A description of any assumptions or corrections, such as tests of normality and adjustment for multiple comparisons
☐	☒	A full description of the statistical parameters including central tendency (e.g. means) or other basic estimates (e.g. regression coefficient) AND variation (e.g. standard deviation) or associated estimates of uncertainty (e.g. confidence intervals)
☐	☒	For null hypothesis testing, the test statistic (e.g. *F*, *t*, *r*) with confidence intervals, effect sizes, degrees of freedom and *P* value noted<br>*Give P values as exact values whenever suitable.*
☒	☐	For Bayesian analysis, information on the choice of priors and Markov chain Monte Carlo settings
☒	☐	For hierarchical and complex designs, identification of the appropriate level for tests and full reporting of outcomes
☒	☐	Estimates of effect sizes (e.g. Cohen's *d*, Pearson's *r*), indicating how they were calculated

Our web collection on statistics for biologists contains articles on many of the points above.

Software and code

Policy information about availability of computer code

Data collection	All code is freely available: https://github.com/MRCToxBioinformatics/eIF5A_hypusination_inhibition_silac v0.1.
Data analysis	Methods of proteomics analysis are detailed in methods section.<br>Cell cycle analysis: FlowJo (v10.10) .<br>Charts/graphs and statistical analysis: Graphpad Prism v 10.6 and Microsoft Excel.<br>Western blot densitometry: LICOR Imagestudio lite v5.2.5.

For manuscripts utilizing custom algorithms or software that are central to the research but not yet described in published literature, software must be made available to editors and reviewers. We strongly encourage code deposition in a community repository (e.g. GitHub). See the Nature Portfolio guidelines for submitting code & software for further information.

Data

Policy information about availability of data

All manuscripts must include a data availability statement. This statement should provide the following information, where applicable:

- Accession codes, unique identifiers, or web links for publicly available datasets
- A description of any restrictions on data availability
- For clinical datasets or third party data, please ensure that the statement adheres to our policy

The mass spectrometry proteomics data generated in this study have been deposited in the ProteomeXchange Consortium via the PRIDE partner repository and are

accessible with the dataset identifier PXD051303.
LATTICeA raw images can be requested from JLQ upon reasonable request. Clinical data from the LATTICeA cohort is restricted, and is held by NHS Greater Glasgow and Clyde Biorepository (clare.orange@ggc.scot.nhs.uk; john.lequesne@glasgow.ac.uk) as custodians. Data access can be requested, and any such request will be reviewed and released under their research ethics committee-approved tissue bank protocols. Requests will be reviewed and approved within 6–8 weeks and will be accompanied by a data sharing agreement detailing the conditions and restrictions of use and publication.

Research involving human participants, their data, or biological material

Policy information about studies with human participants or human data. See also policy information about sex, gender (identity/presentation), and sexual orientation and race, ethnicity and racism.

Reporting on sex and gender	N/A
Reporting on race, ethnicity, or other socially relevant groupings	N/A
Population characteristics	N/A
Recruitment	N/A
Ethics oversight	Lung adenocarcinoma under ethical approval for the use of surplus tissue in research (REC reference: 16/WS/0207) and managed by the NHSGGC Bio-repository and Pathology Tissue Resource.

Note that full information on the approval of the study protocol must also be provided in the manuscript.

Field-specific reporting

Please select the one below that is the best fit for your research. If you are not sure, read the appropriate sections before making your selection.

☒ Life sciences ☐ Behavioural & social sciences ☐ Ecological, evolutionary & environmental sciences

For a reference copy of the document with all sections, see nature.com/documents/nr-reporting-summary-flat.pdf

Life sciences study design

All studies must disclose on these points even when the disclosure is negative.

Sample size	The sample size for all data is included in each figure legend. Statistical analysis was only applied to experiments containing three biologically independent repeats, as is standard in the field.
Data exclusions	No data excluded from the study.
Replication	Indication of whether the data was biological-independent replication or technical replication is included in each figure legend.<br>All data showing means with standard deviation were from at least 3 replicates.<br>Statistical analysis was only carried out on data obtained using 3 biological-independent replicates.
Randomization	Allocation was random.
Blinding	Blinding was not possible as specific compounds needed to be administered to cells, or specific comparisons needed to be made to non-tumour tissue.

Reporting for specific materials, systems and methods

We require information from authors about some types of materials, experimental systems and methods used in many studies. Here, indicate whether each material, system or method listed is relevant to your study. If you are not sure if a list item applies to your research, read the appropriate section before selecting a response.

Materials & experimental systems

n/a	Involved in the study
☐	☒ Antibodies
☐	☒ Eukaryotic cell lines
☒	☐ Palaeontology and archaeology
☒	☐ Animals and other organisms
☒	☐ Clinical data
☒	☐ Dual use research of concern
☒	☐ Plants

Methods

n/a	Involved in the study
☒	☐ ChIP-seq
☐	☒ Flow cytometry
☒	☐ MRI-based neuroimaging

Antibodies

Antibodies used

Western blot Supplementary table 2:
Hypusine (Hpu98) (Creative Biolabs, PABL-582, 1:1000)
eIF5A1 (Abcam, ab32443, 1:1000)
EIF5A1/EIF5A2 (Proteintech, 17069-1-AP, 1:1000)
DOHH (Abcam, ab197587, 1:1000)
DHPS (Abcam, ab190266, 1:1000)
p-eIF2α (Ser51) (Abcam, Ab32157, 1:1000)
eIF2α (Cell Signaling, #9722S, 1:1000)
ATF4 (Cell Signaling, #11815, 1:1000)
ATF4 (Abcam, ab270980, 1:1000)
Phospho-4E-BP1 (Ser65) (Cell Signaling, #9451S, 1:1000)
4E-BP1 (Cell Signaling, #9644S, 1:1000)
Phospho-eEF2 (Thr56) (Cell Signaling, #2331S, 1:1000)
eEF2 (Santa Cruz, sc-166415, 1:1000)
Actin (Sigma-Aldrich, A5441, 1:1000)
β-tubulin (Cell Signaling, #2146, 1:1000)
Ndufs1 (Abcam, ab169540, 1:1000)
TIM44 (Abcam, ab194829, 1:1000)
TOM20 (Cell Signaling, 424065, 1:1000)
SDHA (Abcam, ab14715, 1:1000)
SDHB (Abcam, ab14714, 1:1000)
MRPL37 (Proteintech, 15190-1-AP, 1:1000)
MRPS15 (Abcam, ab242120, 1:1000)
RPS6 (Cell Signaling, #2217S, 1:1000)
RPS25 (Abcam, ab254671, 1:1000)
HRI (Invitrogen, #702551, 1:1000)
PERK (C33E10) (Cell Signaling, #3192S, 1:1000)
PKR (Cell Signaling, #12297S, 1:1000)
GCN2 (Cell Signaling, #3302S, 1:1000)
Puromycin (clone 12D10) (Merck, MABE343, 1:10,000)
Phospho-p38 MAPK (Thr180/Tyr182) (Cell Signaling, #4511S, 1:1000)
p38 MAPK (Cell Signaling, #9212S, 1:1000)
Secondary anti-mouse IgG (H+L) (DyLight 800) (Cell Signaling, #5257, 1:15,000)
Secondary anti-mouse IgG (H+L) (DyLight 680) (Cell Signaling, #5470, 1:15,000)
Secondary anti-rabbit IgG (H+L) (DyLight 680) (Cell Signaling, #5366, 1:15,000)
Secondary anti-rabbit IgG (H+L) (DyLight 800) (Cell Signaling, #5151, 1:15,000)

Validation

All antibodies used in the study are commercial and validation was carried out by the manufacturer. Manufacturer/product codes are provided for all antibodies.
Antibodies for PERK, HRI, PKR, GCN2, OMA1, DELE1, eIF5A, DHPS and DOHH were validated by the use of siRNA knockdown.

Eukaryotic cell lines

Policy information about cell lines and Sex and Gender in Research

Cell line source(s)

A549 (CCL-185), HeLa (CCL-2), MCF7 (HTB-22), MCF10A (CRL-10317) and HT-29 (HTB-38) were obtained from ATCC and authenticated by vendor.
Mesothelioma primary cell lines (7T, 8T, 9T and 13T) were derived/authenticated in previous studies (Chernova et al 2016, Grosso et al 2021) and are available from MesoBank UK.
WT and S51A MEFs were a gift from David Ron.

Authentication

Yes, characterised in Chernova et al 2016, Grosso et al 2021 (references supplied in methods). Commercially available lines authenticated by vendor.
WT and S51A MEFs were validated by the lack of p-eIF2a induction (Supplementary figure 1f).

Mycoplasma contamination	no
Commonly misidentified lines (See ICLAC register)	none

Plants

Seed stocks	NA
Novel plant genotypes	NA
Authentication	NA

Flow Cytometry

Plots

Confirm that:

☒ The axis labels state the marker and fluorochrome used (e.g. CD4-FITC).

☒ The axis scales are clearly visible. Include numbers along axes only for bottom left plot of group (a 'group' is an analysis of identical markers).

☒ All plots are contour plots with outliers or pseudocolor plots.

☒ A numerical value for number of cells or percentage (with statistics) is provided.

Methodology

Sample preparation	Cells trysinised and collected in 10% FBS containing media. For cell death, cell pellet resuspended in annexin buffer with annexin and Draq7 and incubated at room temperature for 20 minutes and then kept on ice until analysis. For cell cycle, cell pellet was fixed in ice cold 70% ethanol in PBS overnight at 4oC. Fixed cells were incubated with either FxCycle Violet DNA stain (4,6-diamidino- 2-phenylindole dihydrochloride) (Thermo Fisher Scientific) in 0.1% BSA/PBS overnight at 4oC; or 20 µg/ml propidium iodide (PI) in 1% Triton X-100/PBS and 0.1 mg/ml RNase A overnight at room temperature.
Instrument	BD LRS Fortessa and BD FACS Canto II
Software	BD FACSDIVA (v9.0.1) and FlowJo (v10.10)
Cell population abundance	10,000 events per sample.
Gating strategy	Cell death gated on Annexin-V FITC / Draq7. Live population of cells were Annexin and Draq7 negative. Cell cycle gated on FxCycle/PI width/height and visualised as a histogram. cell cycle state quantified by Dean-Jett-Fox modelling using Flowjo.

☒ Tick this box to confirm that a figure exemplifying the gating strategy is provided in the Supplementary Information.
